# Sustainable Program Assessment Practices: A Review of the ABET and NCAAA Computer Information Systems Accreditation Process

**DOI:** 10.3390/ijerph182312691

**Published:** 2021-12-02

**Authors:** Saqib Saeed, Abdullah M. Almuhaideb, Yasser A. Bamarouf, Dina A. Alabaad, Hina Gull, Madeeha Saqib, Sardar Zafar Iqbal, Asiya Abdus Salam

**Affiliations:** 1 Department of Computer Information Systems, College of Computer Science and Information Technology, Imam Abdulrahman Bin Faisal University, P.O. Box 1982, Dammam 31441, Saudi Arabia; sbsaed@iau.edu.sa (S.S.); yabamarouf@iau.edu.sa (Y.A.B.); daalabbad@iau.edu.sa (D.A.A.); hgull@iau.edu.sa (H.G.); saiqbal@iau.edu.sa (S.Z.I.); aasalam@iau.edu.sa (A.A.S.); 2 SAUDI ARAMCO Cybersecurity Chair, Department of Networks and Communications College of Computer Science and Information Technology, Imam Abdulrahman Bin Faisal University, P.O. Box 1982, Dammam 31441, Saudi Arabia; amalmuhaideb@iau.edu.sa

**Keywords:** accreditation, program assessment, ABET, NCAAA, information systems, outcome measurement, academic quality

## Abstract

Program outcome assessment is a complex process that demands careful planning and resources in order to accurately assess higher-order thinking skills. A well-defined assessment approach provides detailed insights into program weaknesses and leads to continuous improvement. Whereas a poor assessment approach does not reflect the underlying weaknesses and may result in a useless effort. Furthermore, each accreditation body may have a different recommended outcome measurement approach. As a result, academic institutions may make adhoc choices just to satisfy accreditation requirements rather than designing a sustainable measurement approach. On the other hand, the magnitude of huge tasks for satisfying multiple accreditation bodies results in fatigue and mental stress for academic staff. ABET is a well-known international program accreditation body, and NCAAA is a local accreditation body for academic programs in the Kingdom of Saudi Arabia. In this paper, we have documented that how a sustainable outcome measurement mechanism can be designed to satisfy both ABET and NCAAA requirements. The core contribution of this paper is relevant specifically for academic programs in the Kingdom striving to meet both ABET and NCAAA requirements and is also relevant for all education programs to design an appropriate program assessment approach to ensure a sustainable process to foster better learning among students.

## 1. Introduction

Accreditation has become an important quality assurance tool for higher education institutions not only to improve their processes but also to use as a marketing tool to attract more students and funding. There are many local as well as international accreditation agencies in different geographical regions that accredit institutions or a particular program. The majority of the accreditations can be termed as single-tier accreditation, where a particular criterion needs to be satisfied by aspiring academic institutes/programs and there is no further classification among accredited programs. Due to the recent focus on outcome-based education, an academic program intended for accreditation needs to provide evidence that it has a comprehensive system of measuring the intended outcomes of the program. Student outcome attainment requires that higher-order thinking skills be measured to ensure that students have gained the required mastery of skills to be successful in their future professional careers.

An ideal program assessment approach should be valid, lightweight, and easy to verify, however, many additional constraints are relevant, such as management support, faculty support and intention for continuous improvement [1]. In the case, that the core objective of such program assessment is just to satisfy the accreditation requirements then the intended benefits of quality improvement are not gained and adhoc practices take over rather than a systematic approach. Furthermore, due to institutional policies, an academic program may have to meet the accreditation requirements of multiple accreditation bodies. As a result, the measurement for program assessments will require multiple sets of measurement cycles and approaches resulting in more complexity. Such multiple approaches bring confusion to faculty and as a result, may lower the quality of assessments and relevant higher-order skills may not be measured at required rigor.

In this paper, we present a case study from an Information Systems undergraduate program which is required to satisfy the international accreditation, Accreditation Board of Engineering and Technology (ABET) [2] as well as local accreditation, National Commission for Academic Accreditation and Assessment (NCAAA) [3]. Both accreditation bodies have different recommendations regarding student outcomes and measurement processes and running them side by side was a complex and confusing task. As a result, we have outlined a unified program mapping to satisfy both accreditation requirements and a measurement mechanism to align the activities. The findings of the study are helpful for other similar academic programs that are facing the same dilemma.

The rest of the paper is structured as follows: Section 2 provides related work followed by research methodology in Section 3. Section 4 discusses results and is followed by a discussion and a conclusion in Section 5 and Section 6 respectively.

## 2. Related Work

Oudshoorn et al. have described that recently there is an increase in acquiring ABET accreditation by computing programs. They have outlined a set of guidelines for stakeholders aiming to acquire ABET accreditation [4]. Hossain et al. have highlighted that although the scope of institutional and program accreditation is different, due to their similarities a common assessment approach can complement each other. Based on this, they have compared Middle States Commission of Higher Education (MSCHE) and Accreditation Board of Engineering and Technology (ABET) standards and assessment activities [5]. Irons et al. have carried out a study to better understand the value of professional accreditation in the computer science field [6].

Many researchers have documented their ABET accreditation experience to help other institutions in achieving accreditation requirements. Anwar and Richards have compared the accreditation criteria of ABET and the engineering council, both of which are Washington accord signatories and highlighted similarities and differences among their criteria. They proposed the need for alignment among accredited programs of different signatory bodies of the Washington accord [7]. Bachnak, et al. have highlighted that a deep understanding of accreditation procedures and policies can help an academic program to better prepare for ABET accreditation [8]. Goncharow et al. have proposed a system to map computer science course curriculum with national curriculum standards to help educators to reuse material from a standard repository to ensure alignment with national standards [9]. Rabaa’I et al. have shared their experience of ABET accreditation at the American University of Kuwait by providing assessment results of different performance indicators and student outcomes [10]. Osman et al. have developed a dataset of mapping of program educational objectives and student outcomes from ABET self-study reports of 32 accredited programs and applied different classification techniques to get insights in mapping [11].

Program assessment is one of the critical tasks to verify that an academic program is able to meet the intended student outcomes. Carelli has presented a case study of an academic program’s assessment for acquiring ABET accreditation to provide guidelines for other aspiring institutions to acquire ABET accreditation [12]. Shafi et al. have presented a case study of student outcome assessment of computer science and computer information systems programs based on the successful accreditation experience of ABET [1]. Khan has proposed an assessment approach for program educational objectives and student outcomes for ABET accreditation based on their successful experience in the computer science program and King Abdulaziz University Jeddah, Saudi Arabia [13]. Ahmad and Qahmash have developed eleven critical success factors in pursuit of ABET accreditation and developed their prioritization based on fuzzy analytical hierarchical processing and full consistency method to facilitate institutions in their preparation for ABET accreditation [14]. Rashid has proposed an approach for the preparation of program assessment data for ABET accreditation that provides clarity to faculty members and coordinators about their responsibilities in the accreditation process [15]. Hussain et al. have highlighted the important factors of an outcome-based assessment model to appropriately measure the student outcomes of engineering programs in a longitudinal assessment cycle [16].

Establishing an institutional quality management system can help in fostering quality culture and ultimately help academic programs in achieving accreditation requirements. Almuhaideb and Saeed have proposed a set of best practices to deploy quality assurance practices to foster appropriate outcome-based learning [17]. Furthermore, Almuhaideb and Saeed have also proposed a set of organizational processes to approach ABET accreditation based on their experience of ABET accreditation of Bachelor of Cybersecurity and digital forensics program [18]. Abd El-Aziz et al. have highlighted that how the curriculum of the computer information system program at Jouf University contributed to achieving the program’s educational objectives as well as student outcomes in a systematic manner [19]. Alarifi has shared the experience of a mechanical engineering program accreditation at Majmaah University Saudi Arabia in the context of international (ABET) as well as local (NCAAA) accreditation. The study highlighted the need for an ethical-related course to improve professional responsibility among mechanical engineering students [20].

Since the ABET assessment process is quite complex and modern technologies have the potential to improve the data collection and reporting process. Therefore, Sabir et al. have developed an application in Microsoft access to facilitate data management of program assessment for ABET accreditation [21]. Alhakami et al. have used different data mining algorithms to predict student performance in attaining student outcomes based on assessments conducted in course files [22]. Similarly, Schahczenski and Van Dyne have developed a software tool to facilitate program assessment data collection and analysis which reduced the efforts required to collect and maintain program assessment data by faculty [23].

Recently, like all other fields’ accreditation activities are also impacted by the COVID-19 pandemic. Hussain et al. have proposed a digital quality management system for program assessment to facilitate virtual accreditation visits due to the COVID-19 pandemic. This model was applied to three engineering programs, and they recommended its usage by academic institutions and accreditation bodies in remote accreditation processes [24]. Karimi and Manteufel have documented the challenges of the virtual ABET accreditation process due to the COVID-19 pandemic and provided recommendations for the preparation of accreditation documents for such virtual ABET visits [25]. Since conducting lab experiments is a difficult task in online learning, Mohamed et al. [26] have provided a design method to emulate power engineering labs in online learning due to the COVID-19 pandemic. In their model, they provide a simulated environment based on textbook examples and discussion that how the experiment contributed to relevant ABET student outcomes.

Despite these studies, there is no systematic study that provides detailed insights on developing a program assessment strategy that is aligned with different accreditation requirements. Keeping this in view, in this paper we provide a detailed insight into how a program assessment strategy can be formulated which is in line with accreditation requirements of different accreditation bodies.

## 3. Materials and Methods

The findings of this research are part of a longitudinal action research project, initiated to develop sustainable quality practices for computing academic programs, which are aligned with international and national standards. In this contribution, our core research question was that how to define a sustainable program assessment methodology that is aligned with multiple accreditation requirements. In order to answer this question, we adopted a case study approach in our research design. The case study approach is widely used in research studies to provide a rich description of settings to relate research findings in diverse contexts. Our case study is based on a Bachelor of Science in Computer Information Systems (CIS) program. The quantitative data were collected using multiple direct and indirect assessments including surveys and student performance data. To ensure data accuracy, we employed multiple reviews by different team members and during the analysis phase, we conducted a descriptive analysis of assessment data. The findings of this case study will help other academic institutions to align their teaching strategies and assessments with different local and international accreditation requirements.

**Figure 1:**
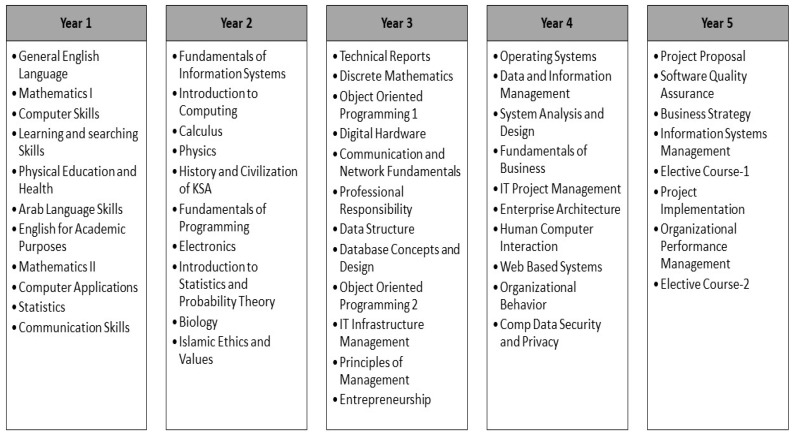
Curriculum of Bachelor of Science in Computer Information Systems Program.

Our case setting (CIS academic program) is offered by the College of Computer Science and Information technology (CCSIT) at Imam Abdulrahman Bin Faisal University (IAU) [27]. Since its inception in 2010, the college has been trying to design an innovative academic program to meet the market needs. Currently, CCSIT offers four undergraduate programs namely, Computer Information Systems, Computer Science, Cybersecurity & Digital Forensics and Artificial Intelligence. Since the university offering the CIS program is NCAAA-accredited, all the academic programs in the university need to follow NCAAA guidelines in program design and assessments, while at the same time aligning themselves to ABET criteria (due to ABET program accreditation). The Bachelor of Science in Computer Information Systems (CIS) program is a five-year program consisting of 152 credit hours. Year 1 is managed by the preparatory year at the Deanship of Preparatory and Supporting Studies before joining CCSIT. During the preparatory year, students are streamed into three academic tracks: health, engineering, and science. The students who are interested in joining CCSIT need to join the science track. The courses that students attend as part of the preparatory year contribute to their Cumulative Grade Point Average (CGPA) at the end of the bachelor’s degree. After completion of their preparatory year, the students start their studies at CCSIT, where Year 2 and Year 3 are common for all students aiming to join any undergraduate program at CCSIT. After the completion of Year 3, students select the respective degree program, and in this paper, we are particularly focusing on the CIS program. Figure 1 shows the curriculum for the CIS program, which has an integration of University, College and Program requirement courses. CIS program aims at preparing competent graduates who will be able to review the required system for implementation and how to integrate technology into the organizational processes.

## 4. Program Mapping and Assessment Matrix

CIS program at CCSIT is an ABET-accredited program. The accreditation by ABET is aimed to ensure that education provided through this program meets an acceptable level of quality. It helps in creating goals for institutional self-improvement and to look for self-regulatory alternatives. The program was accredited by ABET in 2018, and it will be assessed for ABET accreditation in 2023.

### 4.1. Student Outcomes and Performance Indicators

Desired characteristics that are expected to be achieved from students at the end of the program are called Student Outcomes (ABET)/Program Learning Outcomes (NCAAA). To design an optimal teaching strategy, we have used a top-down strategy, where firstly we devised the intended learning outcomes of the academic program. ABET refers to the intended learning outcomes to be achieved at the completion of an academic program as Student Outcomes (SOs), whereas NCAAA uses the term Program Learning Outcomes (PLOs). In this paper we will mainly use the term SOs, however, both terms are interchangeable.

ABET has proposed five generic student outcomes required for a computing-related program and one specific student outcome for an information system related degree program which are numbered as 1–6. On the other hand, NCAAA does not have any such specific recommendations, however, they recommend that program learning outcomes need to be classified into three learning domains, Knowledge & understanding, Skills and Values. Furthermore, each domain needs to have at least one program learning outcome mapped to it. When we mapped ABET proposed CIS student outcomes into learning domains of NCAAA, we found that these outcomes are mapped to only Skills and Values domains, and there is no learning outcome at the knowledge & understanding level. Therefore, we have developed an additional student’s outcome for our CIS program in the Knowledge & Understanding domain, targeting the coverage of fundamental concepts and theories in the information system domain. Since ABET has numbered its student outcomes as 1–6, so to avoid confusion we numbered the additional student outcome as 0, whereas the NCAAA recommends numbering program learning outcomes with a prefix of the respective domain, so CIS program has 1 SO (K1) for Knowledge & Understanding domain, four SOs (S1, S2, S3, S4) for Skills domain and 2 SOs (V1, V2) for Values domain in our CIS program.

Following are the CIS program Student Outcomes/program learning outcomes (labeled with associated learning domain) which satisfy ABET revised criteria for the CIS program, as well as are aligned with NCAAA requirements.

- Define fundamental concepts and theories from information systems and related fields. [Knowledge & understanding]
- Analyze a complex computing problem and apply principles of computing and other relevant disciplines to identify solutions. [Skills]
- Design, implement and evaluate a computing-based solution to meet a given set of computing requirements in the context of the program’s discipline. [Skills]
- Communicate effectively in a variety of professional contexts. [Skills]
- Recognize professional responsibilities and make informed judgments in computing practice based on legal and ethical principles. [Values]
- Function effectively as a member or leader of a team engaged in activities appropriate to the program’s discipline. [Values]
- Demonstrate an understanding of processes that support the delivery and management of information systems within a specific application environment. [Skills]

To ensure that all aspects of a student outcome are covered in our program and required higher-order skills are integrated into the curriculum, each student outcome is further decomposed into performance indicators which are mapped with the entire curriculum. ABET recommends measuring performance indicators whereas NCAAA is only concerned with SOs/PLOs, therefore, in our program mapping we have considered both of them. Following are key performance indicators (PIs) (labeled with associated student outcomes) of the CIS program.

1. Students demonstrate the ability to understand the fundamental concepts related to information system discipline. [SO:0]
2. Students demonstrate the ability to understand the knowledge of supporting disciplines appropriate to the needs of the program. [SO:0]
3. Students demonstrate the ability to decompose a task into appropriate components. [SO:1]
4. Students demonstrate the ability to solicit and formulate requirements specifications. [SO:1]
5. Students demonstrate the ability to estimate the resources required for the proposed solution. [SO:1]
6. Students demonstrate the ability to design a secure computer-based system, process, component, or program to meet desired needs. [SO:2]
7. Students demonstrate the ability to develop a computer-based solution. [SO:2]
8. Students demonstrate competency in creating and executing test cases. [SO:2]
9. Students demonstrate the ability to write technical reports. [SO:3]
10. Students demonstrate the ability to deliver oral presentations. [SO:3]
11. Students demonstrate the ability to learn new skills and apply them rationally to solve the given problem. [SO:4]
12. Students demonstrate knowledge of professional, ethical, legal, social issues and responsibilities. [SO:4]
13. Students demonstrate the ability to produce quality deliverables and meet the deadlines of the group. [SO:5]
14. Students demonstrate the ability to organize themselves and function as a team. [SO:5]
15. Students demonstrate the ability to understand information system management issues, tools and technology. [SO:6]
16. Students demonstrate the ability to evaluate the applicability of a technology or its impact, on an organizational environment. [SO:6]
17. Students demonstrate the application of business knowledge to facilitate the delivery and management of Information Systems. [SO:6]
18. Students demonstrate the ability to manage security risks affecting business continuity. [SO:6]

### 4.2. Designing Teaching Strategy

**Figure A1:**
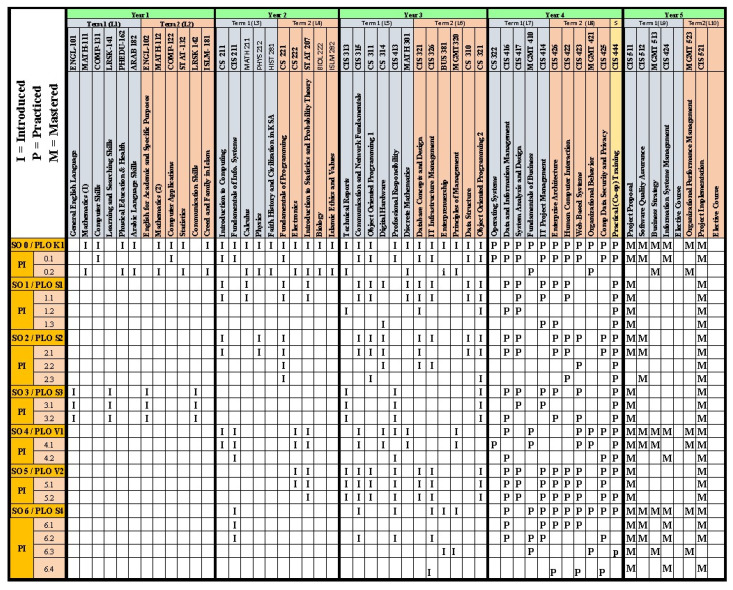
Unified Program Mapping.

As a next step, a program study matrix was developed, so as already mentioned ABET is interested in SOs as well as PI attainment whereas NCAAA is mainly interested in SO attainment. Therefore, we designed a teaching strategy for each performance indicator, each course was mapped to relevant PIs as Introduced (I), Practiced (P) and Mastered(M). Courses from the first three common years were mapped as (I), fourth-year courses were mapped as (P) and final year relevant courses were mapped as (M). To ensure the mapping was correct, each course learning outcome (CLO) that was contributing to a particular PI was documented. As an example, if a particular course’s CLO is mapped to performance indicator 1.1, then it is shown as (SO: 1; PI: 1.1), highlighting that this CLO is mapping to PI 1.1 and SO1. Once all PIs are mapped to the entire curriculum, a mapping for respective CLOs was made, in case any of the PI under a particular SO is mapped to a course then we used the same mapping symbol (I/R/E) for that SO, e.g., for SO 1, a particular course is mapped as “I” for any of the PI 1.1/1.2/1.3, then we will map the SO as “I” as well. In this way, we have a program mapping for all SOs and all PIs. For ABET, we used program mapping at the PI level whereas for NCAAA documentation PI mapping was ignored and only program mapping was used at the SO level. In this way rather than maintaining two different program mapping, we developed a unified program mapping which is shown in Figure A1 in Appendix A. The development of this program mapping was managed by the program quality unit where they coordinated the teaching faculty with each course so that appropriate teaching strategies can be formulated. In our earlier experience, it has been found that if the mapping is left to a few individuals, then the mapping can be developed quickly but it lacks acceptance across faculty and may miss out on important concepts. Furthermore, it was ensured that the curriculum is fully aligned with the Association of Computing Machinery (ACM) [28] and Associational of Information Systems (AIS) [29] joint curriculum guidelines and ABET criteria [2,30].

As a next step, we identified optimal teaching strategies required to teach the curriculum content required for a CLO. As a result, we developed a set of teaching strategies being used in the entire program for each SO, as shown in Table A1 in Appendix A.

### 4.3. Assessment Strategy

In the context of the measuring approach, there were also considerable differences in the requirements of both accreditation bodies. NCAAA requires that the CLOs of each course be measured and then as per program mapping the data of each SO is calculated by averaging all the relevant CLOs. This is very exhaustive and requires that the CLO of each course in the curriculum be considered. As each course has developed an exam blueprint to demonstrate how each CLO is going to be evaluated in that course, so at the completion of each course CLO data is collected from the teaching team which reflects the student performance levels for each CLO in the respective course. Therefore, to collect program assessment data in the case of NCAAA, all the relevant CLO data, contributing to a SOs based on program mapping were averaged from the last two-year course. Only the last two years of the course were considered to collect program assessment data because in the first three years it is not possible to track students only belonging to a particular degree program, as students select the degree at the completion of year three only. In an earlier contribution, we have discussed the processes to have an end of term presentation where each instructor presents the result of CLO attainments and continuous improvement actions are planned based on the feedback of the entire department [17].

On the other hand, for ABET, there is no such restriction, and it is possible to select representative courses to select for formative and summative assessment, so we have developed an assessment plan for ABET as well. In the assessment plan, 4th-year courses are selected for formative assessments only and 5th-year courses are selected for summative assessments. These courses are selected based on the relevance of the course to the PI requirements. For each PI, three courses for assessments were identified, one for formative assessment and two for summative assessments. The assessment plan was approved by the department in the first week of the term. This ensured that the instructors of all identified courses for program assessments know before the commencement of classes that their course will be used for ABET data collection. The rubrics of all performance indicators were defined which were also approved by the department. Attainment of SOs was collected using summative data, formative data, alumni, faculty and exit surveys. Each course selected for formative or summative assessment had a dedicated comprehensive question targeting the measuring PI in the final exam as per the defined rubric. These assessment results were shared with the quality unit and quality unit members collaborated with the instructor team in filling the summative form. In the case of surveys, relevant surveys had relevant questions relating to PIs.

### 4.4. Assessment Data

At the completion of the semester, each instructor filled an excel sheet and this data was collected by the program quality unit which aggregated the data to prepare the student performance for each SO. In order to ensure the validity of data peer reviews were conducted at course level data, as well as program level data.

The data collection for ABET was a little complex, here for each course selected for a given PI, the student performance was categorized into four categories named Poor, Developing, Developed and Exemplary based on the defined rubrics. To ensure that assessment data covers the required rigor of higher-order thinking skills, during the data collection process program quality unit members collaborated with faculty. Initially, they conducted a workshop with faculty that how higher-order thinking skills can be measured and how rubrics were applied. Later during the data collection process, they supported in identifying students’ performance levels and data collection. This collaborative approach helped especially the new faculty with how good assessments can be designed to appropriately measure the analytical skills required by the performance indicators. All this assessment data was collected in an excel sheet for each program and peer review by program quality unit members ensured the data validity. Furthermore, random checks were conducted for PI data to backtrack the data to the course level to ensure the correctness of data. Finally, complete data was presented to the department board for verification and approval. The data was analyzed using cohort analysis, where formative and summative data helped to understand the student learning progress through their academic journey. Furthermore, the results of formative and summative data were correlated.

## 5. Results

The attainment was calculated based on the number of students reached to developed and exemplary categories and an attainment target of 70% was set. Formative and summative data was documented starting from each course belonging to a PI and then the average results of each PI and then the average for each SO is calculated. Similarly, faculty, exit, and alumni survey attainment was documented separately at each PI level and averaged at the SO level. Since survey questions use a Likert scale from 1–5, so we use the attainment formula as (Strongly Agree + Agree + (1/2 of Sometimes True) percentages). Table 1 provides attainment data for each assessment tool.

**Figure 2:**
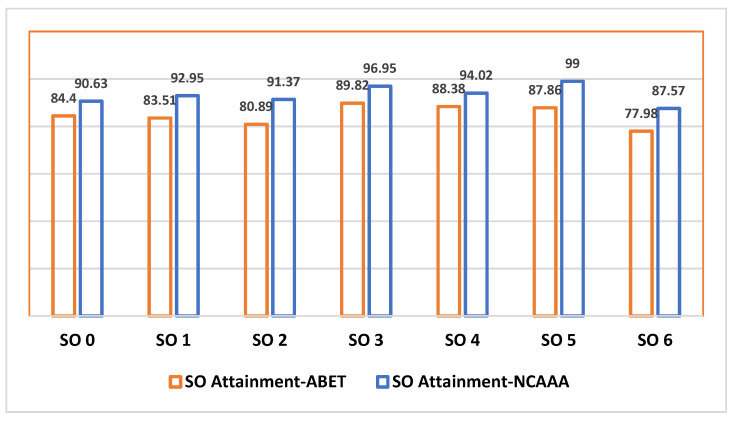
ABET and NCAAA SO Attainment Results.

Finally, as shown in Figure 2, all the data is aggregated at the SO level based on NCAAA as well as ABET measurements approaches. It should be noted that such average value is mainly for calculation purposes, while preparing the continuous improvement action plan, the CIS program not only relied on the attainment values but also drilled down at each course level, PI and SO level and also taken into consideration feedback given in surveys.

**Figure 3:**
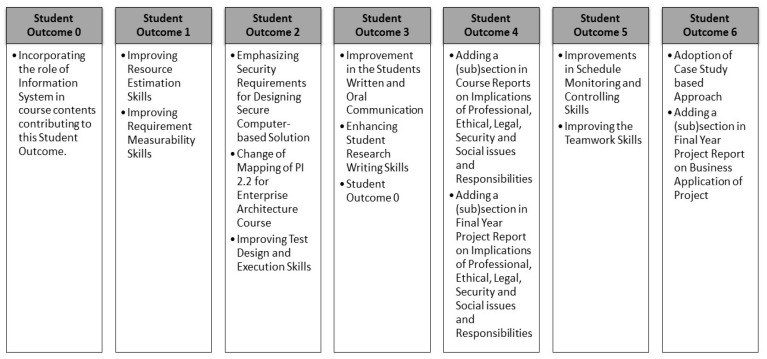
Continuous Improvement Action Plan.

Based on this extensive program assessment exercise, a continuous improvement action plan was formulated which is shown in Figure 3.

## 6. Analysis

Although NCAAA and ABET program assessments had considerable differences, ABET data was based on selective courses whereas NCAAA approach was more exhaustive covering all courses, but still the results in Figure 2 do not highlight many variations, as the variation threshold is almost the same for all student outcomes. We also highlight that a sustainable assessment approach requires faculty motivation and support to manage the workload of data collection and analysis [31,32]. Especially in the scenarios where such multiple accreditations are targeted there is a need for dedicated staff to manage the entire process with extensive collaboration with faculty members. As future work, we propose to develop prototypes that can support data measurement and also can apply rubrics on assessments to generate better results, e.g., automated scoring of written essays [33]. However, for such a system to be useful, it must be properly developed and aligned with user practices [34,35].

## 7. Discussion

Establishing a sustainable assessment mechanism requires that the assessment approach is understandable to all stakeholders. In the case of multiple accreditation requirements, it is important to have a unified program mapping to avoid confusion and assessment methods should be overlapped wherever possible to minimize effort and stress on academic staff [31,32]. As Hossain et al. [5] highlighted that institutional and program assessment activities can be merged so our findings highlight that even program accreditation efforts can be combined by developing a unified program mapping and assessment strategy resulting in one continuous improvement action plan. As Rashid [20] highlights a clarity of assessments process to faculty members results in better acceptance, so we observed that such a unified approach, as well as collaboration by quality unit members, facilitated program assessments. Furthermore, early alignment of curriculum with ACM, AIS and ABET guidelines ensured the coverage content as per the program’s requirements, thus going beyond Goncharow et al.’s approach [9] where they proposed a national level curriculum repository for standardizing course curriculum.

We also highlight that selection of courses from where the data should be collected in one assessment cycle should be based on the relevance of the course to the PI requirements, rather than ease in data collection. In each cycle, there should be a change in the assessed courses wherever possible to balance the load among course instructors. There is a tradeoff between the reliability of data and the effort required to collect data. Selecting all PIs data from a few courses results in less effort but may compromise the quality of data, therefore, it needs to be ensured that there is a reasonable number of courses identified for data collection in one cycle.

Instructors need to ensure that the assessment questions aimed at measuring ABET SOs/PIs are in line with the rigor required in rubrics. The questions in these assessments should strictly follow the rubrics designed for PIs. The development of unified rubrics, training workshops and collaboration of a central quality unit are the main hallmarks of success to ensure that assessment practices are targeting the required higher-order skills.

## 8. Conclusions

To ensure compliance with accreditation procedures, academic programs need to document the attainment of student outcomes. The precision of the student attainment process is vital to draw continuous improvement action plans. In many countries, there are local accreditation bodies and international accreditation bodies and normally there are differences in their approach to program assessment. In this paper, we have presented that how the computer information systems program solved this challenge by devising an assessment approach that is aligned with international accreditation (ABET) and local accreditation (NCAAA). The findings will help other academic institutions facing a similar situation to appropriately design program outcome measurement mechanisms.

## Figures and Tables

**Table 1:** Attainment data of Performance Indicators.

Performance Indicator	Formative Assessment	Summative Assessment	Exit Survey	Faculty Survey	Alumni Survey	Overall
PI 0.1	97.1	98.275	83.97	92	77	89.67
PI 0.2	79.42	67.865	85.34	86	77	79.13
PI 1.1	76.92	100	89.66	89	77	86.52
PI 1.2	75.63	94.075	87.94	92	82.5	86.43
PI 1.3	43.66	97.035	84.21	86	77	77.58
PI 2.1	57.96	86.29	85.34	79.5	82.5	78.32
PI 2.2	98.82	94.08	86.22	86	71.5	87.32
PI 2.3	88.97	57.685	84.48	88	66	77.03
PI 3.1	100	98.89	89.66	91	67	89.31
PI 3.2	99.27	94.82	87.07	88	82.5	90.33
PI 4.1	97.92	95.32	87.93	88.5	82.5	90.43
PI 4.2	89.13	76.775	86.21	85	94.5	86.32
PI 5.1	100	98.89	88.79	92	66	89.14
PI 5.2	92.96	98.885	87.07	87.5	66.5	86.58
PI 6.1	70.59	88.045	85.34	92	83.5	83.9
PI 6.2	64.52	90.505	84.49	89	66	78.9
PI 6.3	74.2	57.15	85.34	81.5	55	70.64
PI 6.4	99.99	81.425	80.45	75.5	55	78.47

## Data Availability

The data presented in this study are available on request from the corresponding author. The data are not publicly available due to institutional requirements.

## Appendix A

**Table A1:** Learning Outcome and Teaching Strategies Alignment.

Program Learning Outcomes/Student Outcomes	Teaching Strategies
Define fundamental concepts and theories from information systems and related fields.	Lectures, Videos, Class Discussion, Lab Work, Group Discussion, Case Studies, Assignments
Analyze a complex computing problem and apply principles of computing and other relevant disciplines to identify solutions.	Lectures, Real World Scenarios discussion, Experiments, Case Studies, Design and Development exercises, Group Projects, group discussion, project-based problem solving, Hands-on practice, Brainstorming sessions, Lab-quizzes
Design, implement and evaluate a computing-based solution to meet a given set of computing requirements in the context of the program’s discipline.	
Communicate effectively in a variety of professional contexts.	
An understanding of processes that support the delivery and management of information systems within a specific application environment.	
Recognize professional responsibilities and make informed judgments in computing practice based on legal and ethical principles.	Graduation project, Group projects, Design and development of exercises, group discussion
Function effectively as a member or leader of a team engaged in activities appropriate to the Program’s discipline.	Group projects, Role Playing in a team, Self-reflection and assessment exercises, individual and group presentations, Essay writing, Presentations, Research-based report writing, Reading exercises
